# Pillar 3: Does banking regulation support stakeholders’ interest in banks financial and risk profile?

**DOI:** 10.1371/journal.pone.0258449

**Published:** 2021-10-27

**Authors:** Anna Pilková, Michal Munk, Ľubomír Benko, Petra Blažeková, Jozef Kapusta

**Affiliations:** 1 Comenius University in Bratislava, Bratislava, Slovakia; 2 Constantine the Philosopher University in Nitra, Nitra, Slovakia; 3 University of Pardubice, Pardubice, Czech Republic; 4 Pedagogical University of Cracow, Kraków, Poland; Szechenyi Istvan University: Szechenyi Istvan Egyetem, HUNGARY

## Abstract

The paper examines the interest of the commercial banks’ stakeholders in Pillar 3 disclosures and their behaviour during the timing of serious market turbulence. The aim is to discover to which extent current banking regulation supports stakeholders’ interest in the information required by regulators to be disclosed. The examined data consists of log files that were pre-processed using web mining techniques and from which were extracted frequent item sets by quarters and evaluated in terms of quantity. The authors have proposed a methodology to evaluate frequent item sets of web parts over a dedicated time. Based on the verification of applied methodology on two commercial banks, the results show that stakeholders’ interest in disclosures is highest in the first quarter at each year and after turbulent times in 2009 their interests decreased. Moreover, the results suggest that stakeholders expressed higher interest than in regulatory required Pillar 3 information in the following group of information: Pillar3 related information, Annual reports, Information on Group. Following our results, the paper contributes to cover the gap in the research by analysing Pillar 3 disclosures and their compliance with regulatory requirements, which also increase the interest of the relevant stakeholders to conduce them as an effective market discipline tool.

## Introduction

In the recent post-financial crisis years, significant changes have occurred in financial markets regulation and supervision. The financial institutions face challenges as reduction of risk-taking in line with tightening of regulatory requirements and the increase of regulatory directives and guidelines published by the European authorities. These directives cover primarily risk areas related to capital, liquidity, credit, counterparty exposures, market, operational and securitisation. They aim to eliminate systemic risk through supervision and more importantly to reinforce market discipline [1].

Market discipline as one of the three pillars of the stable financial market (the other two are regulation and supervision) has been in regulatory interest since 2001. However, the issuance of Basel II and Basel III regulations has built in market discipline into the regulatory framework through Pillar 3 standards. The process of final Pillar 3 tuning is still ongoing, and its main focus is focused on the methodological changes, feedback from related parties and broad discussions aimed to reach enhancement and reinforcement of the market discipline [2,3].

Topics of Pillar 3 and disclosures have also attracted researchers. They have focused mainly on weaknesses of the disclosures (mainly huge costs, ineffective implementation) and less on benefits [4–6]. Research on stakeholders’ interest and comments towards Pillar 3 disclosures is very limited. The situation is even worse in commercial banks operating in Central and Eastern European countries where their owners are big international financial groups located in developed countries. Evidence is in the paper Munk et al. [7] according to which the relevance of the published Pillar 3 information is not in particular interest of stakeholders, especially information related to mandatory Pillar 3 disclosures. All in all, both from regulatory status and research focus is evident that Pillar 3 framework as a supervisory market discipline tool still does not fulfil its main goal to bring adequate information to key market participants to effectively enhance market discipline. Therefore, consolidation of further research, theory and regulation is crucial for its reinforcement. Following these results our research focuses on studying stakeholders’ behaviour and how and to which extent stakeholders of the commercial banks use Pillar 3 disclosed information.

### Research objective and assumptions

The paper aims to examine the website data dedicated to Pillar 3 disclosures of commercial banks operating in CEE country to study the behaviour of stakeholders concerning the timing of serious market turbulence.

The article summarizes the results of previous research focused on stakeholders’ interest in Pillar 3 information during the years 2009–2015. In the current research, we focus on verifying our latest findings, especially in connection with verifying the continuation of the established trend characterized by the low interest of stakeholders in Pillar 3 information.

We have made the following assumptions:

We assume the continuation of the established trend after the gradual fading of the consequences of the financial crisis from 2012–2015, which is characterized by a lack of interest in Pillar 3 information.

We assume that the behaviour of stakeholders to the published Pillar 3 information will no longer be reflected in any time trend or seasonality.

We will verify the assumptions on data of the accesses of the website of another equally important bank operating in Slovakia during the years 2016–2018.

The rest of the paper is structured as follows. The next section describes the related work in the field of market discipline and Pillar 3 disclosures. The third section summarizes the applied methodology to obtain the Results. The fourth section is focused on the results of the web usage analysis and interpretation of the results. Subsequently, the last section provides the conclusion and contribution of the research to improve Pillar 3 regulatory aims and increase stakeholders’ interests in Pillar 3 regulatory information.

## Literature review

Pillar 3 represents the regulators’ tool for market discipline enhancement by meaningful, relevant, and transparent disclosures. In the last two decades, the regulators’ goal is to conduce them as an effective market discipline tool, which fulfils regulators’ expectations in the areas of standardization, consistency, comparability, and transparency. To bring the stability of the financial markets, the coherence among market discipline, regulatory requirements and supervision is required, as this consistency reduces the potential for regulatory arbitrage. However, there are some depressive effects of implementing regulation, such as reduction in competition, innovation, and profitability and increase in regulatory change costs [8]. On the contrary, these extensive reporting requirements imposed after the global financial crisis support a more efficient, stable, inclusive financial system and are safer than before the financial crisis [9,10]. Based on our reviewed studies, which assess compliance of the Pillar 3 disclosures with regulatory requirements we obtained the following findings. First of all, there is a lack of standardization of the disclosures, which is important due to its impact on the banks’ performance indicators. Disclosures were found general, providing no further useful information, infrequent, annual disclosures were repetitive and symbolic [11], there is an imbalance among some disclosure categories and lack of standardization due to data scarcity [12]. Furthermore, disclosures in their prescriptive nature do not provide an adequate risk picture as they are predominantly backwards-looking. The delivery of the understanding and relevant information to stakeholders is impeded by a dynamic of the risk events [13]. Thus, we agree with the authors that there is an opportunity for directors as stakeholders to decide the provision of additional information to supplement Pillar 3 disclosures and support the market discipline mechanism. Moreover, Savvides and Savvidou [14] point out that the level of harmonization is far from regulators’ intentions because the banks do not disclose risk information in a consistent, easily accessible, or usable manner. Supervisors need to advocate informative risk disclosures, which should be reviewed and updated regularly.

Moreover, the level of the quality of the risk disclosures influences also Pillar 3 content efficiency. Additionally, the overall quality of risk disclosures is poor based on Kabir and Sobhani’s study [15], which analysed the level of risk disclosures of the 46 banks between 2009 and 2013. This is in conjunction with Barakat and Hussainey’s study [16], which concludes that the quality of disclosures also depends on the ownership structure of the bank. These authors propose to achieve better risk reporting quality through various channels: more active audit committee, lower executive ownership, operating under regulations promoting bank competition. Moreover, Khalil and Alam [17] propose an enhancement of market discipline by the introduction of the independent risk disclosure council (composing of representatives of regulators, banks, investors). The council could determine the minimum standard for risk disclosure and the utilization of the information disclosed in the periodic accounts and annual reports, which also reduces the cost of supervision.

Importantly, a balance in transparency should be held as the aggressive decrease in the risk-taking by increasing disclosures can hinder economic growth. This negative effect of market discipline implementation should be closely monitored, as a market discipline plays a vital part in risk-taking behaviour of commercial banks [18]. The role of market discipline is important in evading financial crisis and has come out even stronger in this endeavour because information asymmetry led to moral hazard in commercial banks. Accordingly, there is an assumption that banks do not regard the content of the disclosures in turbulent times. The results of Kabir and Sobhani’s [15] study (period from 2009 to 2013) revealed mimicking tendency of the banks to disclose in the same standard as competitors, in a conventional prescribed format of reporting risk with no explanation of significant risk factors.

Substantially, the beneficial effects of Pillar 3 disclosures are supported (revealed) by loads of research studies. Pillar 3 improves the safety of the banking system [19], offers banks to raise cheaper capital [20], decreases information asymmetry [21–23], quarterly reporting is useful to investors [22] and improves the ex-ante risk sharing provided by financial intermediation [24]. Additionally, the increased risk disclosures brought liquidity benefits when Pillar 3 became effective and its compliance is enforced by the banking regulator [25]. The level of supervisory power positively influences the disclosure of Pillar 3 requirements, specifically on loan loss provisions [26]. Moreover, in the assessment of efficiency of Pillar 3 disclosures the interest of key parties in the content of the disclosures is important. In a few studies, the authors highlight these categories with high content relevancy for investors, which are: credit risk and liquidity risk [27], market risk [28]. De Araujo and Leyshon’s study [29] reveals a window in content relevancy of disclosures as depositors and creditors are most responsive to information such as the bank’s assets, off-balance sheet items, and ratings for other banking activities. Stakeholders value the quantitative information more than the qualitative one and can positively influence bank risk-taking [30]. Additionally, website disclosures are a timely disclosure medium and rich form of communication available to a broad range of stakeholder groups [31]. Therefore, the sensitive response of the stakeholders to the negative disclosures can also trigger runs on an inefficient bank [32] and can lower the distance to default [33]. Furthermore, Oliveira et al. [34] found that stakeholder perceptions in case of manipulation of risk disclosures may be influenced by the reputational benefit of the bank’s management. While investors and customers welcome increased disclosure, care must be taken to ensure that adequate value is being derived from the disclosed information.

In addition, there are rare studies assessing Pillar 3 information disclosures in CEE countries from stakeholders’ perspectives and usage information by them. This type of research is crucial for effective supervisory market discipline implementation. There are arising issues about the specific factors concerning Pillar 3 disclosures, such as content relevancy, timing, and the compliance of the Pillar 3 disclosures with regulatory requirements to serve as an effective market discipline tool [35–37]. Additionally, according to a few studies that analyse the interest of the stakeholders in Pillar 3 disclosures, the most visited parts of the disclosures were not solo Pillar 3 information required by regulators but information on Group, which owns the bank and Pillar 3 information together with Annual reports [7] or Emitent Prospects [38].

The study contributes to cover the research gap by deep analysis of the interest of the stakeholders in Pillar 3 disclosures in relation to market turbulences based on website data of commercial banks in CEE countries. The study is also dedicated to analyzing Pillar 3 disclosures’ compliance with regulatory requirements, which also increase the interest of the relevant stakeholders to serve as an effective market discipline tool.

## Materials and methods

The used methodology is based on the procedures and results of the research project VEGA 1/0776/18 (Optimizing the content and structure of Pillar 3 disclosures based on modelling their use by commercial bank’s stakeholders), where particular attention is on web usage mining. Since Basel regulations are complex systems, detecting the problems of their lack of efficiency requires the most accurate analysis and application of non-traditional approaches such as web mining. This research project serves as a basis for the methodology of this study, and we try to identify potential problems in the usage of bank portals by analysing behaviour patterns of web users following the required information from Pillar 3.

### Methodology

**Fig 1:**
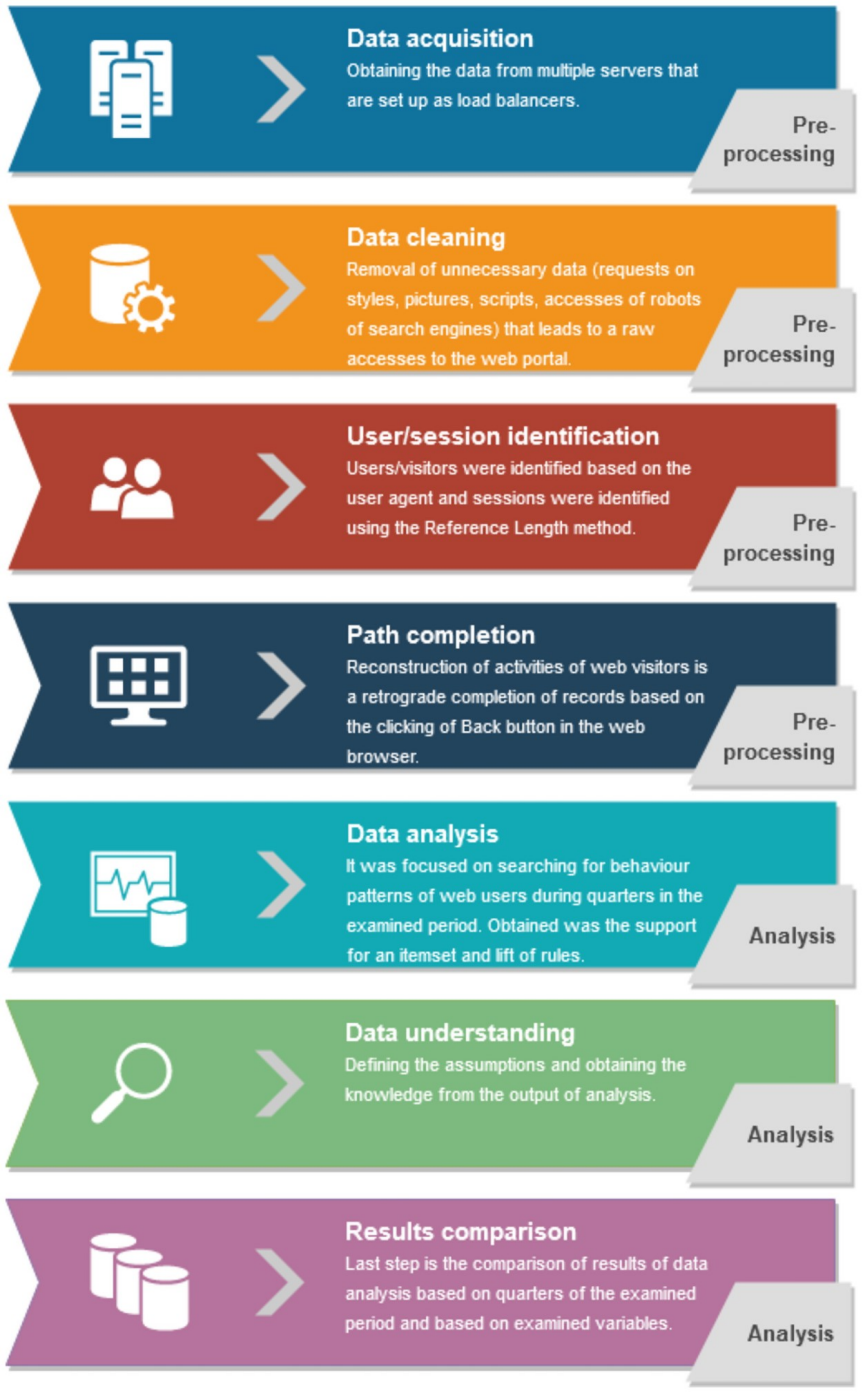
The approach used to process the log files.

The research methodology was inspired by Munk et al. [39] and it was done the same way as in Munk et al. [7] where it was used to evaluate the frequent item sets in terms of quantity. In this web usage analysis were data related to Pillar 3 obtained from the web server log files of domestic significant commercial banks operating in Slovakia. In comparison to the previous research, another bank was used with the idea to validate the results of previous research of Munk et al. [7]. The webserver log files keep information about visitors, which can be used for the analysis of visitor’s behaviour. Data pre-processing of their usage consists of data cleaning, integration, transformation, session identification, path completion and data reduction [40,41]. Data preparation was done based on Munk et al. [39] where it was needed to pre-process log files from multiple servers that are used as load balancers and the used methods were inspired by Pamutha et al. [42] and Spiliopoulou et al. [43]. The web usage analysis was realised on a sample of log accesses which were obtained after the data pre-processing included data cleaning, session identification and path completion. The applied methodology is depicted in Fig 1 and is based on Munk et al. [7] research.

### Dataset composition

This article is based on the results of the research by Munk et al. [7] that dealt with the analysis of website data dedicated to Pillar 3 disclosures of commercial banks. Therefore, two web server log files of domestic significant commercial banks operating in Slovakia were used. The first log file used in the research of Munk et al. [7] contained more than 10 000 000 log accesses that were obtained after data pre-processing. These accesses represent the five years 2009–2015 where the year 2009 represents the year of the financial crisis. On the other hand, the years 2010–2015 represent the years after the financial crisis. The second log file used in this research comes from a different web server. The log file consisted of more than 35 000 000 log accesses that were obtained after data pre-processing. The examined period was of years 2016–2018. During the data pre-processing phase were created various variables. This way time variables were created for both log files that represented the quarter of the specific year. Three variables were added to the dataset—Quartal, Year and YearQuartal which represents the number of quartal of the year. Also, a variable Category was created to unite the accessed the web parts of web portal into web categories. The composition of both created datasets from the log files are described in Table 1 that contains information about the analysed web categories. The analysed banks provided different levels of content based on the website taxonomy of the surveyed banks (Table 1). Although the web categories of the surveyed banks are at different levels of detail, in the case of both banks we clearly identified web parts with Pillar 3 content (in accordance with the regulator’s requirements). This allowed us to monitor the time trend and seasonality in stakeholder behaviour in relation to Pillar 3 information.

**Table 1:** Website taxonomy.

**First analysed bank 2009–2015**		
/About bank/	/Pillar 3 disclosure requirements/	/Pillar3 Q-terly Info/
/About bank/	/Pillar 3 disclosure requirements/	/Pillar3 Semiannualy Info/
/About bank/	/Pillar 3 related/	/Rating/
/About bank/	/Pillar 3 related/	/Annual Reports/
/About bank/	/Pillar 3 related/	/Group/
/About bank/	/Pillar 3 related/	/Information for Banks/
/About bank/	/Pillar 3 related/	/Emitent Prospects/
/About bank/	/Pillar 3 related/	/General Shareholder Meeting/
/About bank/	/Pillar 3 related/	/Financial Reports/
/About bank/	/Other/	
**Second analysed bank 2016–2018**		
/Pillar 3/	/Pillar 3 disclosure requirements/	
/Pillar 3/	/Pillar 3 related/	
/Other/	/Products and services for customers/	
/Other/	/Information service/	
/Other/	/About us/	
/Other/	/Press centre/	
/Other/	/Social responsibility/	
/Other/	/Documents/	

## Results

The experiment analysed website data dedicated to Pillar 3 disclosures of two commercial banks operating in Slovakia and studied the behaviour of stakeholders in relation to the timing of serious market turbulence. The aim is to find out whether current banking regulation supports stakeholders’ interest in the information required by the regulator to be disclosed and confirm the research assumptions. The first subsection is a summary of the previous research done by Munk et al. [7]. A log file from the years 2009–2015 was analysed and based on the results were established the hypotheses for further research. The second subsection is focused on website data collected during the years 2016–2018. The data is dedicated to Pillar 3 disclosures of the second commercial bank operating in Slovakia and examined was the behaviour of stakeholders to their interest in Pillar 3 disclosures.

### Analysis of the stakeholders’ behaviour and their interest in pillar 3 disclosure during turbulent times (2009–2015) in the Slovak commercial bank

Munk et al. [7] analysed accesses to the website of a commercial bank operating in Slovakia that was focused on Pillar 3 disclosures. The authors recommend further changes in commercial bank’s information disclosure for obtaining an effective market discipline mechanism. The results served as a stepping-stone for our further research presented in the following subsection. For that reason, the most important conclusions were described in this subsection. The web portal was divided into the following parts, which were subsequently analysed: */Group/*, */Annual Reports/*, */Rating/*, */Emitent Prospects/*, */General Shareholder Meeting/*, */Financial Reports/*, */Information for Banks/*, */Pillar 3 Semiannually Information/*, */Pillar 3 Quarterly Information/*. The analysis was done on five-year data 2009–2015 and key findings are summarized according to the years below.

In the first quarter of 2009 (Table 2), the most visited were web parts */Group/*, */Pillar3 Q- terly Info/*, */Rating/*, */Annual Reports/*, */Information for Banks/*, */Pillar3 Semiannualy Info/*, */Emitent Prospects/*. The pairs identified (Table 3) with high interest were (*/Group/*, */Pillar3 Q-terly Info/*), (*/Annual Reports/*, */Pillar3 Q-terly Info/*) and others (Table 3). Positive correlation was identified for (*/Emitent Prospects/*, */Pillar3 Semiannualy Info/*). In the first quarter of 2010 (Table 2), the web parts */Group/*, */Pillar3 Q-terly Info/*, */Annual Reports/*, */Rating/* were the most visited parts. The most visited pair (Table 3) of the web parts was (*/Pillar3 Q-terly Info/*, */Annual Reports/*). Web parts pairs (*/Group/*, */Rating/*) and (*/Pillar3 Q-terly Info/*, */Rating/*) were pairs with less interest. The highest degree of positive correlation was identified for (*/General Shareholder Meeting/*, */Pillar3 Q-terly Info/*), (*/Pillar3 Semiannualy Info/*, */Pillar3 Q-terly Info/*) and (*/Pillar3 Q-terly Info/*, */Annual Reports/*).

**Table 2:** Support: Web part traffic rate.

*min support = 1%*	09Q1	10Q1	11Q1	12Q1	13Q1	14Q1	15Q1
*/Group/*	58.48	38.89	50.00	45.83	48.08	45.76	46.23
*/Pillar3 Q-terly Info/*	36.77	37.04	28.57	20.83	25.00	24.26	23.68
*/Rating/*	30.12	22.22	17.86	16.67	17.31	16.32	16.98
*/Annual Reports/*	25.87	31.48	21.43	16.67	19.23	17.43	15.99
*/Information for Banks/*	23.89	11.11	7.14	< 1	3.85	< 1	< 1
*/Pillar3 Semiannualy Info/*	17.42	3.70	< 1	8.33	3.85	< 1	< 1
*/Emitent Prospects/*	15.15	< 1	< 1	< 1	< 1	< 1	< 1
*/General Shareholder Meeting/*	< 1	1.85	< 1	12.50	5.77	5.64	< 1
*/Financial Reports/*	< 1	< 1	< 1	< 1	< 1	< 1	< 1

Note: Marked supports are > 15%.

**Table 3:** Support: Web part pair traffic rate.

*min support* = 1%	09Q1	10Q1	11Q1	12Q1	13Q1	14Q1	15Q1
(*/Group/*, */Pillar3 Q-terly Info/*)	18.90	3.70	< 1	8.33	3.85	< 1	< 1
(*/Annual Reports/*, */Pillar3 Q-terly Info/*)	18.80	22.22	17.86	8.33	13.46	13.78	14.38
(*/Rating/*, */Group/*)	16.66	7.41	3.57	< 1	< 1	< 1	< 1
(*/Pillar3 Q-terly Info/*, */Pillar3 Semiannualy Info/*)	16.32	3.70	< 1	4.17	< 1	< 1	< 1
(*/Rating/*, */Information for Banks/*)	16.27	< 1	< 1	< 1	< 1	< 1	< 1
(*/Rating/*, */Annual Reports/*)	15.93	1.85	< 1	< 1	< 1	< 1	< 1
(*/Group/*, */Information for Banks/*)	15.78	< 1	3.57	< 1	< 1	< 1	< 1
(*/Rating/*, */Pillar3 Q-terly Info/*)	15.54	5.56	< 1	< 1	< 1	< 1	< 1
(*/Annual Reports/*, */Information for Banks/*)	15.00	1.85	< 1	< 1	< 1	< 1	< 1
(*/Annual Reports/*, */Group/*)	14.59	3.70	< 1	4.17	< 1	< 1	< 1
(*/Pillar3 Q-terly Info/*, */Emitent Prospects/*)	14.57	< 1	< 1	< 1	< 1	< 1	< 1
(*/Pillar3 Q-terly Info/*, */Information for Banks/*)	14.18	1.85	< 1	< 1	< 1	< 1	< 1
(*/Annual Reports/*, */Pillar3 Semiannualy Info/*)	14.05	1.85	< 1	4.17	< 1	< 1	< 1
(*/Annual Reports/*, */Emitent Prospects/*)	13.64	< 1	< 1	< 1	< 1	< 1	< 1
(*/Group/*, */Pillar3 Semiannualy Info/*)	13.62	< 1	< 1	4.17	< 1	< 1	< 1
(*/Group/*, */Emitent Prospects/*)	13.56	< 1	< 1	< 1	< 1	< 1	< 1
(*/Rating/*, */Pillar3 Semiannualy Info/*)	13.45	< 1	< 1	< 1	< 1	< 1	< 1
(*/Rating/*, */Emitent Prospects/*)	13.36	< 1	< 1	< 1	< 1	< 1	< 1
(*/Emitent Prospects/*, */Pillar3 Semiannualy Info/*)	13.24	< 1	< 1	< 1	< 1	< 1	< 1
(*/Information for Banks/*, */Emitent Prospects/*)	13.23	< 1	< 1	< 1	< 1	< 1	< 1
(*/Information for Banks/*, */Pillar3 Semiannualy Info/*)	12.98	< 1	< 1	< 1	< 1	< 1	< 1
(*/Pillar3 Q-terly Info/*, */General Shareholder Meeting/*)	< 1	1.85	< 1	< 1	< 1	< 1	< 1

Note: Marked supports are > 5%.

In the first quarter of 2011 (Table 2), the web part */Group/* was the most visited web part with the highest interest. The other web parts with high interest were */Pillar 3 Q- terly Info/*, */Annual Reports/*, */Rating/*. The pair (Table 3) with the high level of interest was (*/Pillar3 Q-terly Info/*, */Annual reports/*) and a positive correlation was detected for this pair too. Similar behaviour was identified for the year 2012 (Table 2) as in the previous years. The highest support was detected for */Group/*, */Pillar3 Q-terly Info/*, */Rating/*, */Annual Reports/*. The pairs (Table 3) with the highest visits were (*/Pillar3 Q-terly Info/*, */Group/*) and (*/Pillar3 Q-terly Info/*, */Annual Reports/*). The highest correlation was detected for (*/Annual Reports/*, */Pillar 3 Semiannualy Info/*), (*/Pillar3 Q-terly Info/*, */Annual Reports/*) and (*/Pillar 3 Semiannualy Info/*, */Pillar3 Q-terly Info/*).

In 2013 (Table 2), the most visited web part was */Group/*. The other very interesting parts were */Pillar3 Q-terly Inf/*, */Annual Reports/* and */Rating/*. The pair (Table 3) identified with high interest was (*/Pillar3 Q-terly Info/*, */Annual Reports/*). The highest correlation was detected for (*/Annual Reports/*, */Pillar3 Q-terly Info/*) and (*/Pillar3 Q-terly Info/*, */Group/*). In the first quarter of 2014 and 2015 (Table 2), the most visited web parts were */Group/*, */Pillar3 Q-terly Info/*, */Annual Reports/*, */Rating/*. The pair (Table 3) identified with high interest was (*/Pillar3 Q-terly Info/*, */Annual Reports/*) and also positive correlation was detected for this pair.

#### Results summary

The results highlighted that stakeholders were interested in Pillar 3 disclosures (regulatory and accounting) mainly in the first quarter. Stakeholders’ interest was not in Pillar 3 information as a solo, but together with *Annual reports* and *Information on Group*. Moreover, the interest in the disclosed information decreased after turbulent times in 2009. The global financial crisis has had a significant impact on the increased interest in disclosure and related information. This manifested itself in seasonality, i.e. in the increased interest in Pillar 3 information in the first, respectively in the second quarter in the first years (2009–2011) after the end of the financial crisis (2009: *Q* = 8.258, *df* = 3, *p* = 0.0410; 2010: *Q* = 12.581, *df* = 3, *p* = 0.0056; 2011: *Q* = 11.539, *df* = 3, *p* = 0.0091). In the following years (2012–2015) not only did the interest in Pillar 3 information decrease but there was no time trend (*p* > 0.05) and no seasonality in the interest of stakeholders in this information (2012: *Q* = 4.154, *df* = 3, *p* = 0.2453; 2013: *Q* = 3.255, *df* = 3, *p* = 0.3539; 2014: *Q* = 4.565, *df* = 3, *p* = 0.2066; 2015: *Q* = 3.000, *df* = 3, *p* = 0.3916) [7].

### Analysis of the stakeholders’ behaviour and their interest in pillar 3 disclosures in 2016–2018

**Fig 2:**
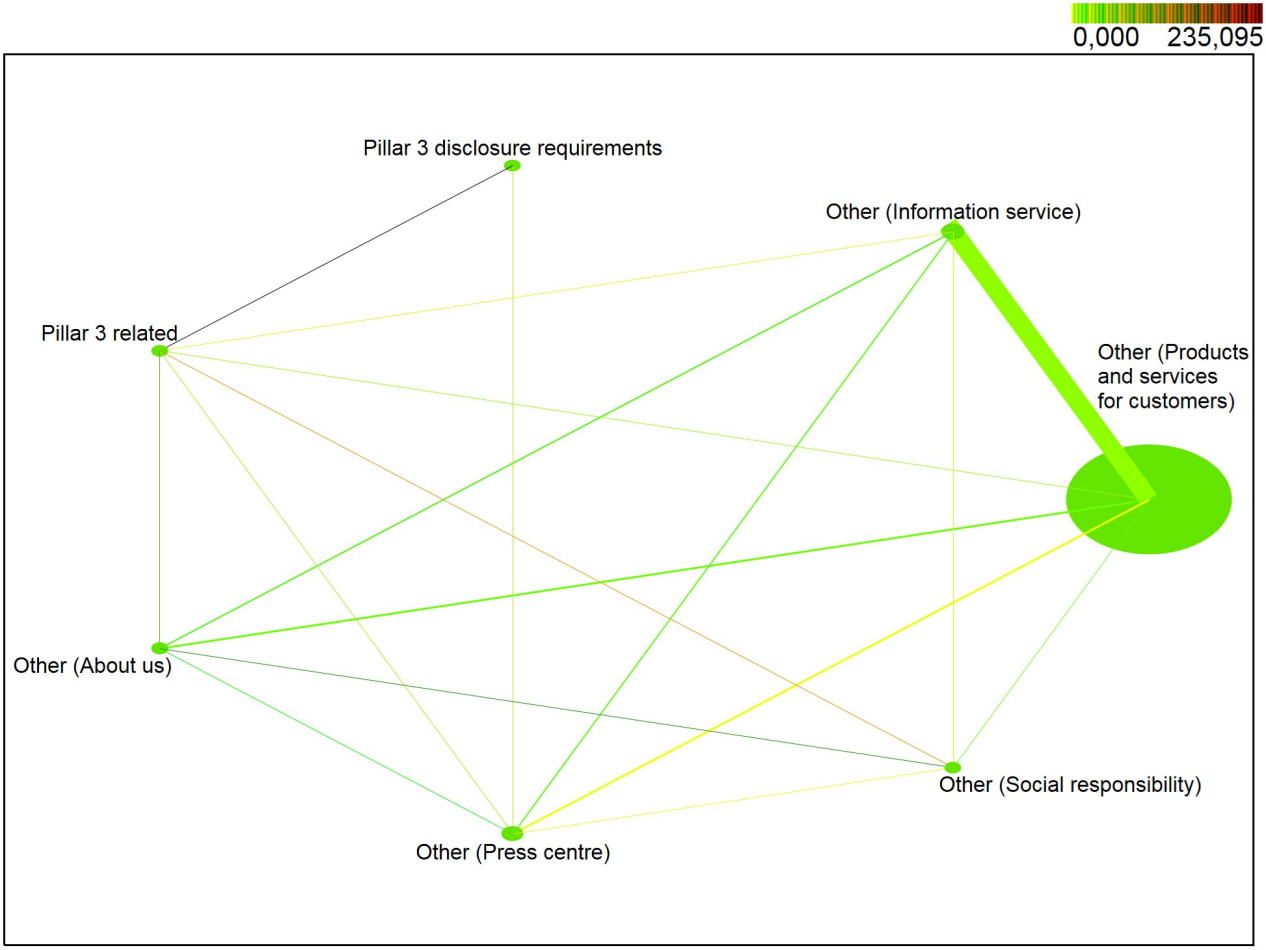
Visualization of frequented web parts of the first quarter of 2018.

In this section, we focus on verifying the continuation of the established trend in the years after the crisis of 2012–2015. The aim of this part is to analyse the behaviour of stakeholders in the period of 2016–2018 in relation to the published Pillar 3 information, in terms of verifying null statistical hypotheses, resulting from established research assumptions that the behaviour of stakeholders in relation to published Pillar 3 information is not influenced by time trend or seasonality. In this part of our research, we analysed website data dedicated to Pillar 3 disclosures of the second commercial bank operating in Slovakia and studied the behaviour of stakeholders in relation to their interest in Pillar 3 disclosures. The website data were divided into eight categories: */Pillar 3 disclosure requirements/*, */Pillar 3 related/*, */Other—About us/*, */Other—Press centre/*, */Other—Information service/*, */Other—Social responsibility/*, */Other—Products and services for customers/*, */Other—Documents/*. During the first quarter of 2018 (Fig 2), the web part */Other—Products and services for customers/* was one of the most visited web parts with 94% *support*. Web parts */Other—Information service/* and */Other—Press Center/* occurred in identified sessions with a probability of about 4%. Web Parts */Other—About us/* and */Pillar3 related/* were less popular with a probability of less than 0.3%. Web parts */Other—Social responsibility/* and */Pillar3 disclosure requirements/* occurred in identified sessions with the *support* of less than 0.1%. The web part */Other—Documents/* did not meet the minimum support, i.e. the probability of occurrence in the identified sessions is less than 0.01%.

In the first quarter of 2018 (Fig 2), pairs (*/Other—Products and services for customers/*, */Other—Information service/*) with approximately 2% *support* were among the most visited pairs of web parts. Other pairs (*/Other—Products and services for customers/*, */Other—Press center/*), (*/Other—Products and services for customers/*, */Other—About us/*) and (*/Other—Information service/*, */Other—About us/*) reached a probability of about 0.14%. Other pairs of web parts achieved a probability of less than 0.08%. The highest level of positive correlation (*lift > 1*) was obtained for the pair (*/Pillar3 related/*, */Pillar3 disclosure requirements/*) with *lift = 235*. The high level was also reached by the pair (*/Other—About us/*, */Other—Social responsibility/*) and (*/Other—Social responsibility/*, */Pillar3 related/*) with *lift* between 100–109. High level of positive correlation was also achieved by positive pair (*/Other—About us/*, */Pillar3 related/*) with *lift* = 86. Positive correlation with *lift* < 10 reached a pair (*/Other—Information service/*, */Other—About us/*), (*/Other—Social responsibility/*, */Other—Information service/*), (*/Pillar3 related/*, */Other—Information service/*), (*/Pillar3 related/*, */Other—Press center/*), (*/Other—Press center/*, */Other—Social responsibility/*), (*/Other—Press center/*, */Pillar3 disclosure requirements/*) and (*/Other—About us/*, */Other—Press center/*). Negative correlation (*lift* < 1) was identified and web parts occur more often separately than together in identified sessions for pairs *(/Other—Press center/*, */Other—Information service/*), (*/Other—About us/*, */Other—Products and services for customers/*), (*/Other—Social responsibility/*, */Other—Products and services for customers/*), (*/Other—Information service/*, */Other—Products and services for customers/*), (*/Pillar3 related/*, */Other—Products and services for customers/*) and (*/Other—Press center/*, */Other—Products and services for customers/*).

*Note*: *NODE SIZE—relative support of each web part, LINE THICKNESS—relative joint support of two web parts, COLOR DARKNESS OF LINE—relative lift of two web parts*.

**Fig 3:**
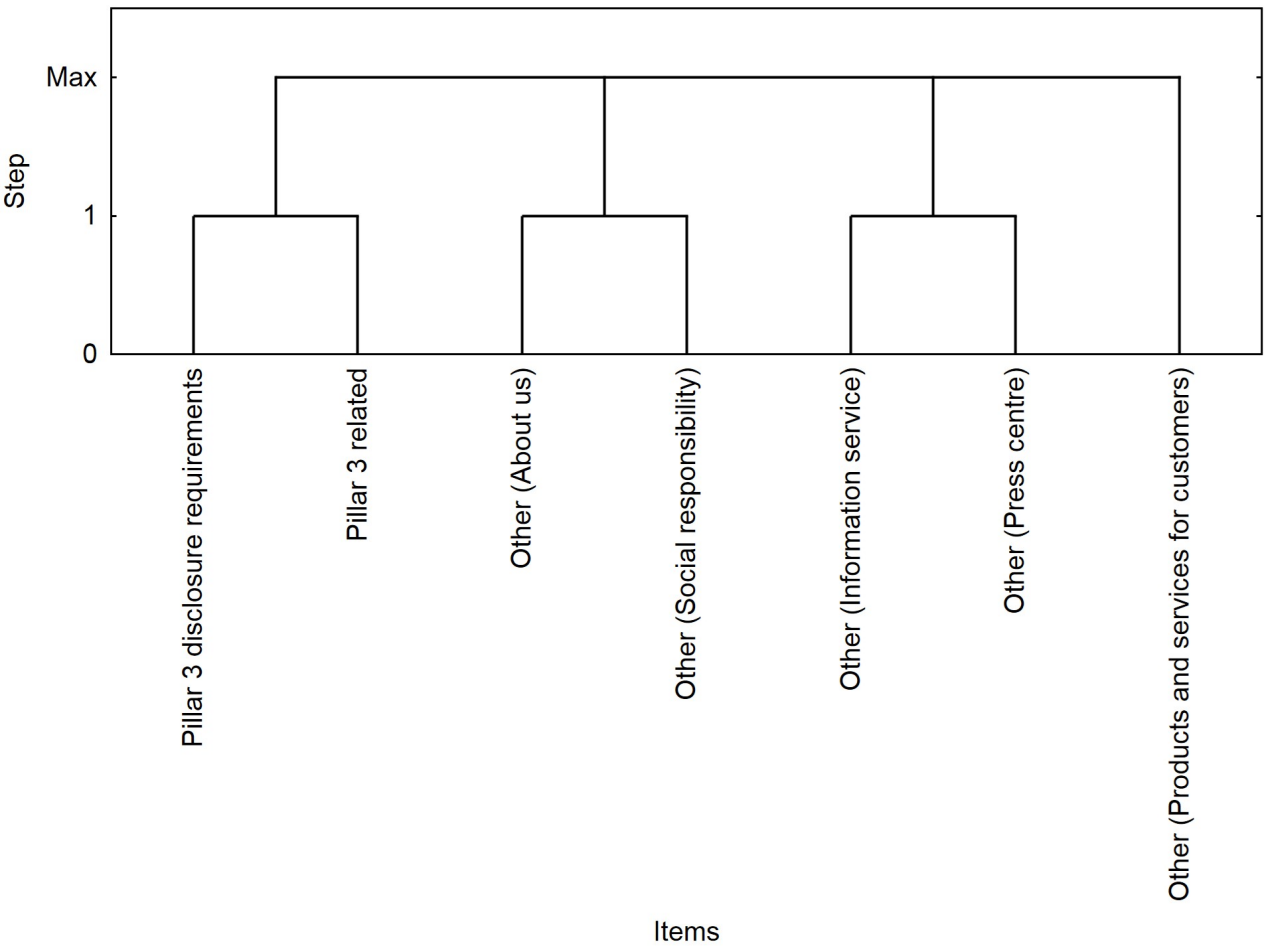
Visualization of segments in the first quarter.

The results of the segmentation (Fig 3) confirm the results of the association analysis for the pairs of web parts (*/Pillar3 disclosure requirements/*, */Pillar3 related/*) and (*/Other—About us/*, */Other—Social responsibility/*), where a positive correlation was identified (*lift* > 1). Part of the sessions was characterized by a visit to both Pillar 3 web parts, where the highest level of interest was achieved (*lift* = 235).

**Fig 4:**
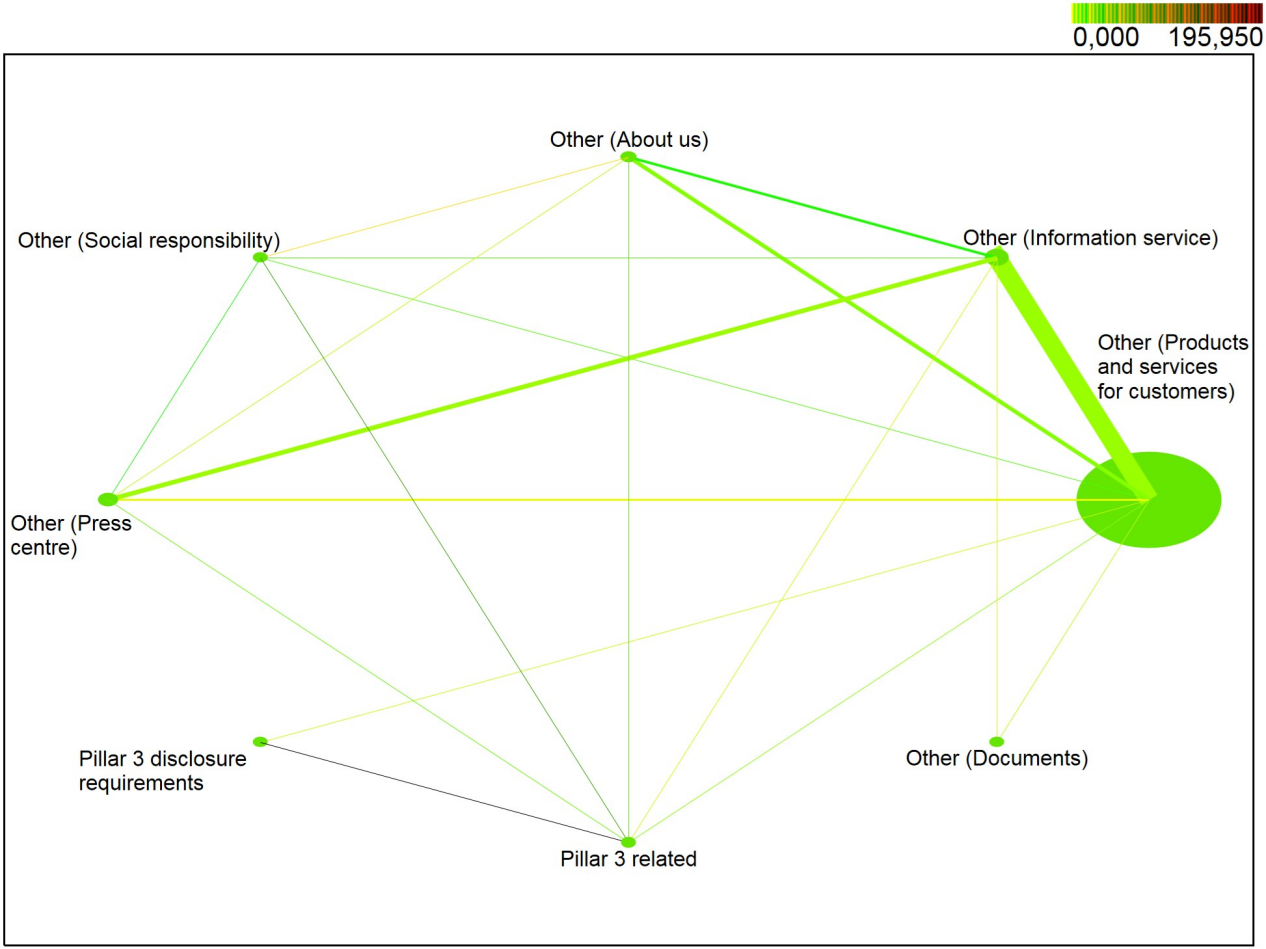
Visualization of frequented web parts of the second quarter of 2018.

In the second quarter of 2018 (Fig 4), the web part */Other—Products and services for customers/* belonged to the most visited web part with the *support* of 92%. The web part */Other—Information service/* occurred in the identified sessions with a probability of more than 6% and the web part */Other—Press centre/* with a probability of about 3%. The web part */Other—About us/* also belonged to the less visited web parts with a probability of just over 1%. The web part */Pillar3 related/* was one of the least visited with *support* of about 0.2%. Other web parts */Other—Social responsibility/*, */Pillar3 disclosure requirements/* and */Other—Documents/* occurred in the identified sessions with a probability of less than 0.1%.

In the second quarter of 2018 (Fig 4), the pair (*/Other—Products and services for customers/*, */Other—Information service/*) with the *support* of almost 2% was among the most visited pairs of web parts. Pairs of web parts *(/Other—Information service/*, */Other—Press center/*), (*/Other—Products and services for customers/*, */Other—About us/*), (*/Other—Information service/*, */Other—About us/*) and (*/Other—Products and services for customers/*, */Other—Press center/*) achieved *support* between 0.2–0.4%. The remaining pairs of web parts achieved the *support* of less than 0.06% but met the requirement for minimum support of at least 0.01%. The highest level of interest was achieved by a pair (*/Pillar3 disclosure requirements/*, */Pillar3 related/*) with *lift* = 196. A high degree of positive correlation was also achieved by a pair of web parts (*/Pillar3 related/*, */Other—Social responsibility/*) with *lift* = 74. The higher interest was also achieved by pairs (*/Other—Social responsibility/*, */Other—About us/*) and (*/Pillar3 related/*, */Other—About us/*) with a lift between 19 and 27. A positive correlation (*lift* > 1) was identified and web parts occur more often together than separately in the identified sessions for (*/Other—Documents/*, */Other—Information service/*), (*/Other—Social responsibility/*, */Other—Information service*/), (*/Pillar3 related/*, */Other—Information service/*), (*/Other—Press center/*, */Other—Social responsibility/*), (*/Pillar3 related/*, */Other—Press center/*), (*/Other—Information service/*, */Other—About us/*) and (*/Other—Press center/*, */Other—Information service/*), which reached a value between 1 and 7. On the other hand, for pairs (*/Other—About us/*, */Other—Products and services for customers/*), (*/Other—Information service/*, */Other—Products and services for customers/*) a (*/Other—Press center/*, */Other—Products and services for customers/*) a negative correlation was identified (*lift* is less than 1).

*Note*: *NODE SIZE—relative support of each web part, LINE THICKNESS—relative joint support of two web parts, COLOR DARKNESS OF LINE—relative lift of two web parts*.

**Fig 5:**
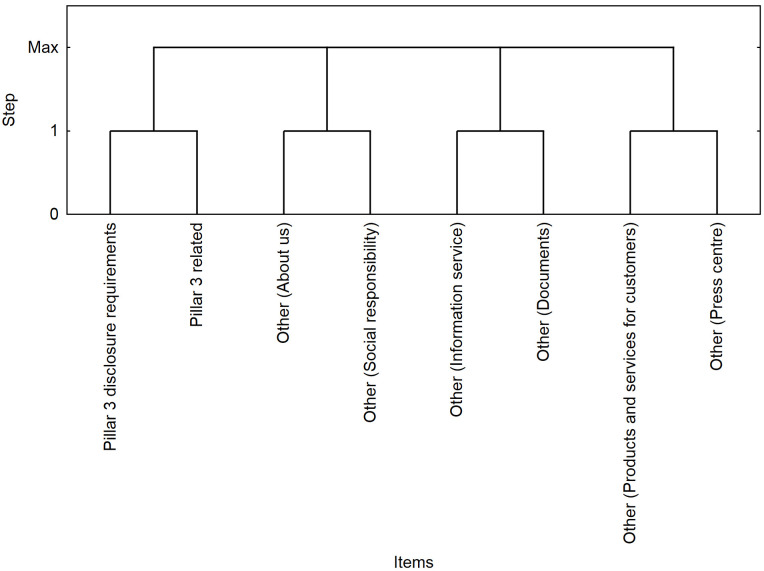
Visualization of segments in the second quarter.

The segmentation outcome (Fig 5) confirms the results of the association analysis for the web part pairs (*/Pillar3 disclosure requirements/*, */Pillar3 related/*), (*/Other—About us/*, */Other—Social responsibility/*) and (*/Other—Information service/*, */Other—Documents/*), where a positive correlation was identified (*lift* > 1). Part of the sessions was characterized by a visit to both Pillar 3 web parts, where the highest level of interest was achieved (*lift* = 196).

**Fig 6:**
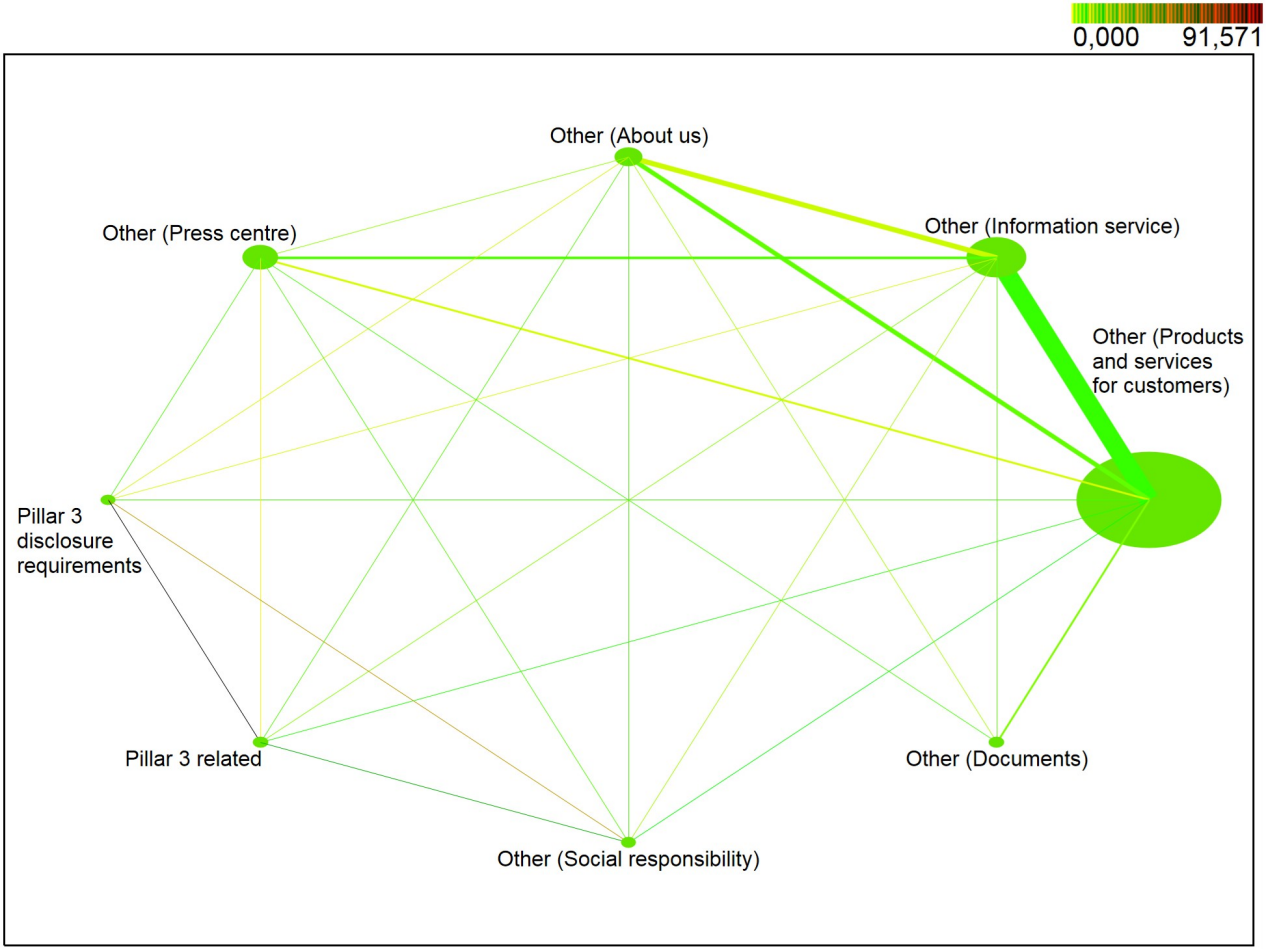
Visualization of frequented web parts of the third quarter of 2018.

In the third quarter of 2018 (Fig 6), one of the most visited web parts was the web part */Other—Products and services for customers/* with 66% *support*. The web part */Other—Information service/* with *support* of more than 22% was also interesting for visitors. Web parts */Other—Press center/* and */Other—About* us*/* were among the less visited with the *support* of 11% and 7%. Less traffic was identified for the web parts */Other—Documents/*, */Pillar3 related/*, */Other—Information Service/*, */Other—Social responsibility/* and */Pillar3 disclosure requirements/* with *support* between 0.2%–0.6%.

In the third quarter of 2018 (Fig 6), the pair (*/Other—Products and services for customers/*, */Other—Information Service/*) was among the most visited web parts with *support* of more than 4%. The pairs of web parts (*/Other—Information Service/*, */Other—About us/*) and (*/Other—Products and services for customers/*, */Other—About us/*) achieved the *support* of around 1%. Less visited pairs (*/*Other—Information Service*/*, */Other—Press center/*), (*/Other—Products and services for customers/*, */Other—Documents/*) and (*/Other—Products and services for customers/*, */Other—Press center/*) achieved *support* between 0.4–0.6%. The other pairs (Fig 6) achieved *support* between 0.01–0.2%. The highest level of interest was achieved by the pair (*/Pillar3 disclosure requirements/*, */Pillar3 related/*) with *lift* = 92. A significant degree of interest was also identified for the pairs of web parts (*/Other—Social responsibility/*, */Pillar3 disclosure requirements/*) and (*/Other—Social responsibility/*, */Pillar3 related/*), with a *lift* between 30–37. Positive correlation was identified for pairs (*/Other—Social responsibility/*, */Other—About us/*), (*/Pillar3 disclosure requirements/*, */Other—About us/*), (*/Pillar3 related/*, */Other—About us/*), (*/Other—Social responsibility/*, */Other—Information Service/*), (*/Other—Social responsibility/*, */Other—Press center/*), (*/Other—Documents/*, */Other—Information Service/*) and (*/Other—Documents/*, */Other—Products and services for customers/*) with a *lift* between 1 and 6. A negative correlation was identified for the other pairs of web parts, that is, they occurred more frequently in the identified sessions separately than together (*lift* <1).

*Note*: *NODE SIZE—relative support of each web part, LINE THICKNESS—relative joint support of two web parts, COLOR DARKNESS OF LINE—relative lift of two web parts*.

**Fig 7:**
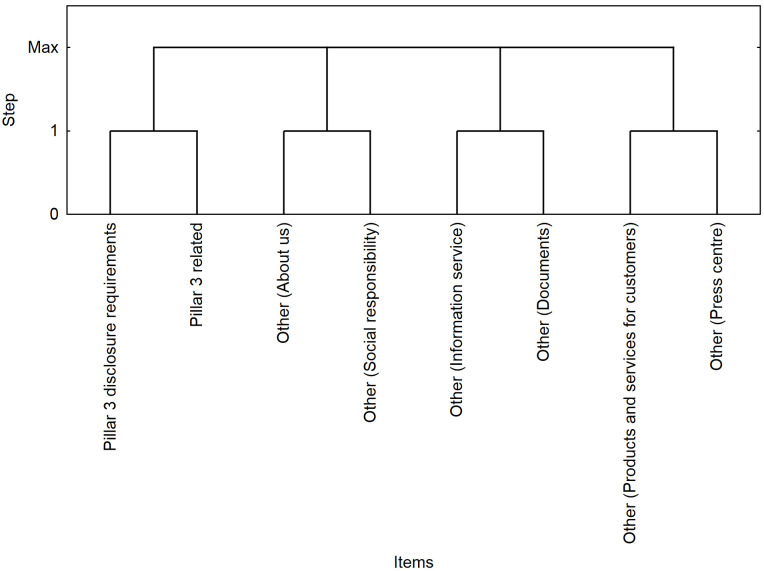
Visualization of seating segments in the third quarter.

The segmentation results (Fig 7) confirm the results of the association analysis for the web part pairs (*/Pillar3 disclosure requirements/*, */Pillar3 related/*), (*/Other—About us/*, */Other—Social responsibility/*) and (*/Other—Information Service/*, */Other—Documents/*), where a positive correlation was identified (*lift* > 1). Part of the sessions was characterized by a visit to both Pillar 3 web parts, where the highest level of interest was achieved (*lift* = 92).

**Fig 8:**
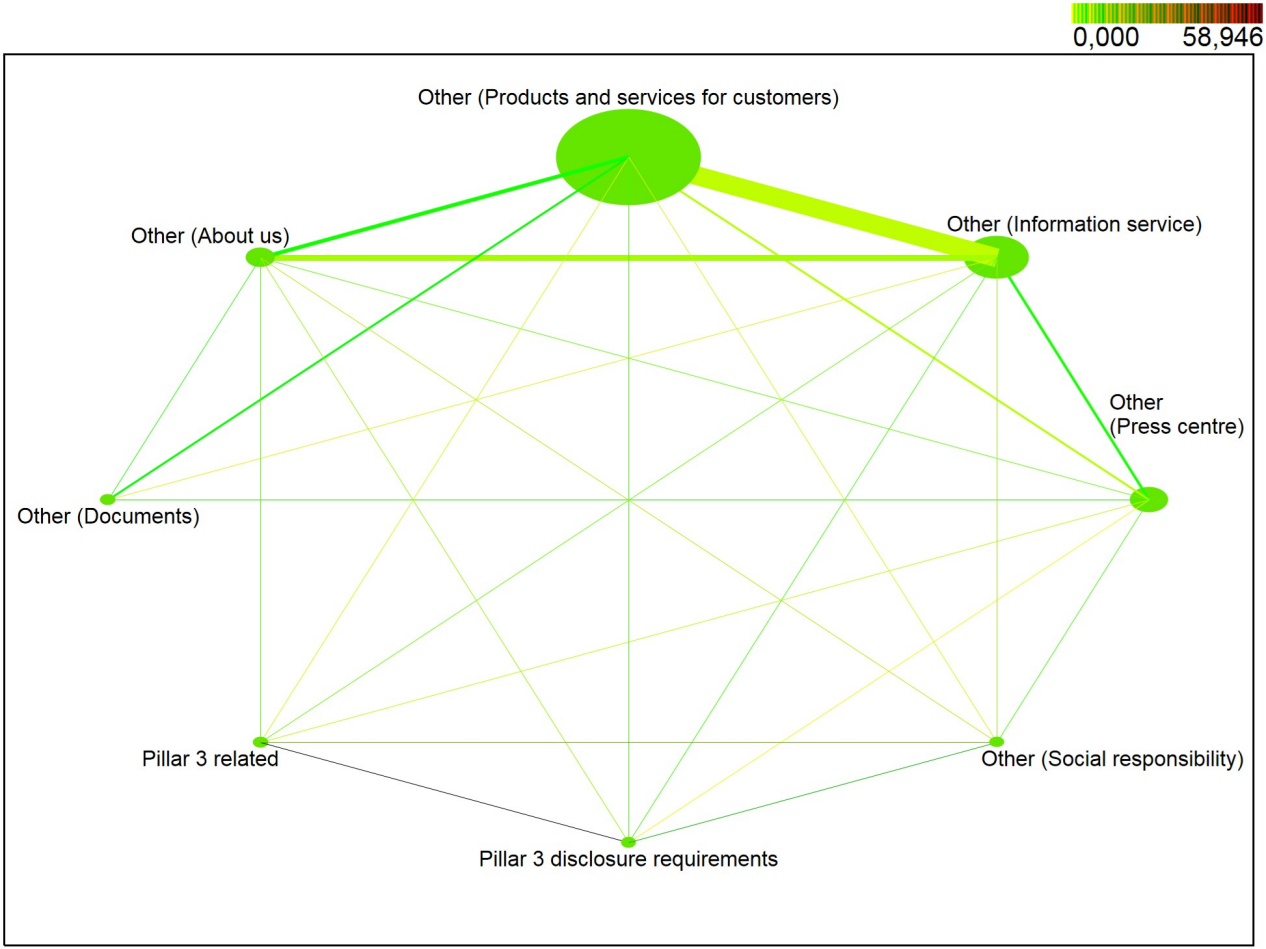
Visualization of frequented web parts of the fourth quarter of 2018.

In the fourth quarter of 2018 (Fig 8), the same behaviour was observed as in the third quarter of the year under study. The most visited web parts included the web part */Other—Products and services for customers/* with 64% *support*. The web part */Other—Information Service/* with *support* of more than 24% was also interesting for visitors. Web parts */Other—Press center/* and */Other—About us/* were among the less visited with the *support* of 11% and 7%. Less traffic was identified for the web parts */*Other—*Documents/*, */Pillar3 related/*, */Other—Information Service/*, */Other—Social responsibility/* and */*Pillar3 disclosure requirements*/* with *support* between 0.2%–0.6%.

A similar trend was also in the case of pairs of web parts, where the results identified in the fourth quarter (Fig 8) are like the third quarter of 2018. The pair (*/Other—Products and services for customers/*, */Other—Information Service/*) was one of the most visited web parts with more *support* of 4%. The pairs of web parts (*/Other—Information Service/*, */Other—About us/*) and (*/Other—Products and services for customers/*, */Other—About us/*) achieved the *support* of around 1%. Less visited pairs (*/Other—Information Service/*, */Other—Press center/*), (*/Other—Products and services for customers/*, */Other—Documents/*) and (*/Other—Products and services for customers/*, */Other—Press center/*) achieved *support* between 0.4–0.6%. The other pairs (Fig 8) achieved *support* between 0.01–0.2%.

*Note*: *NODE SIZE—relative support of each web part, LINE THICKNESS—relative joint support of two web parts, COLOR DARKNESS OF LINE—relative lift of two web parts*.

The highest level of interest in the fourth quarter was achieved by the pair (*/Pillar3 disclosure requirements/*, */Pillar3 related/*) with *lift* = 59. A significant degree of interest was also identified for the pairs of web parts (*/Other—Social responsibility/*, */Pillar3 disclosure requirements/*) and (*/Other—Social responsibility/*, */Pillar3 related/*), with *lift* around 18. Positive correlation was identified for pairs (*/Other—Social responsibility/*, */Other—About us/*), (*/Pillar3 disclosure requirements/*, */Other—About us/*), (*/Pillar3 related/*, */Other—About us/*), (*/Other—Social responsibility/*, */Other—Information Service/*), (*/Other—Social responsibility/*, */Other—Press center/*), (*/Other—Documents/*, */Other—Information Service/*) and (*/Other—Documents/*, */Other—Products and services for customers/*) with a *lift* between 1 and 5. A negative correlation was identified for the other pairs of web parts, i.e. they occurred more frequently in the identified sessions separately than together (*lift* < 1). These results correspond to the behaviour of stakeholders in the third quarter, but in the fourth quarter, a lower level of interest was achieved for the examined pairs of web parts.

**Fig 9:**
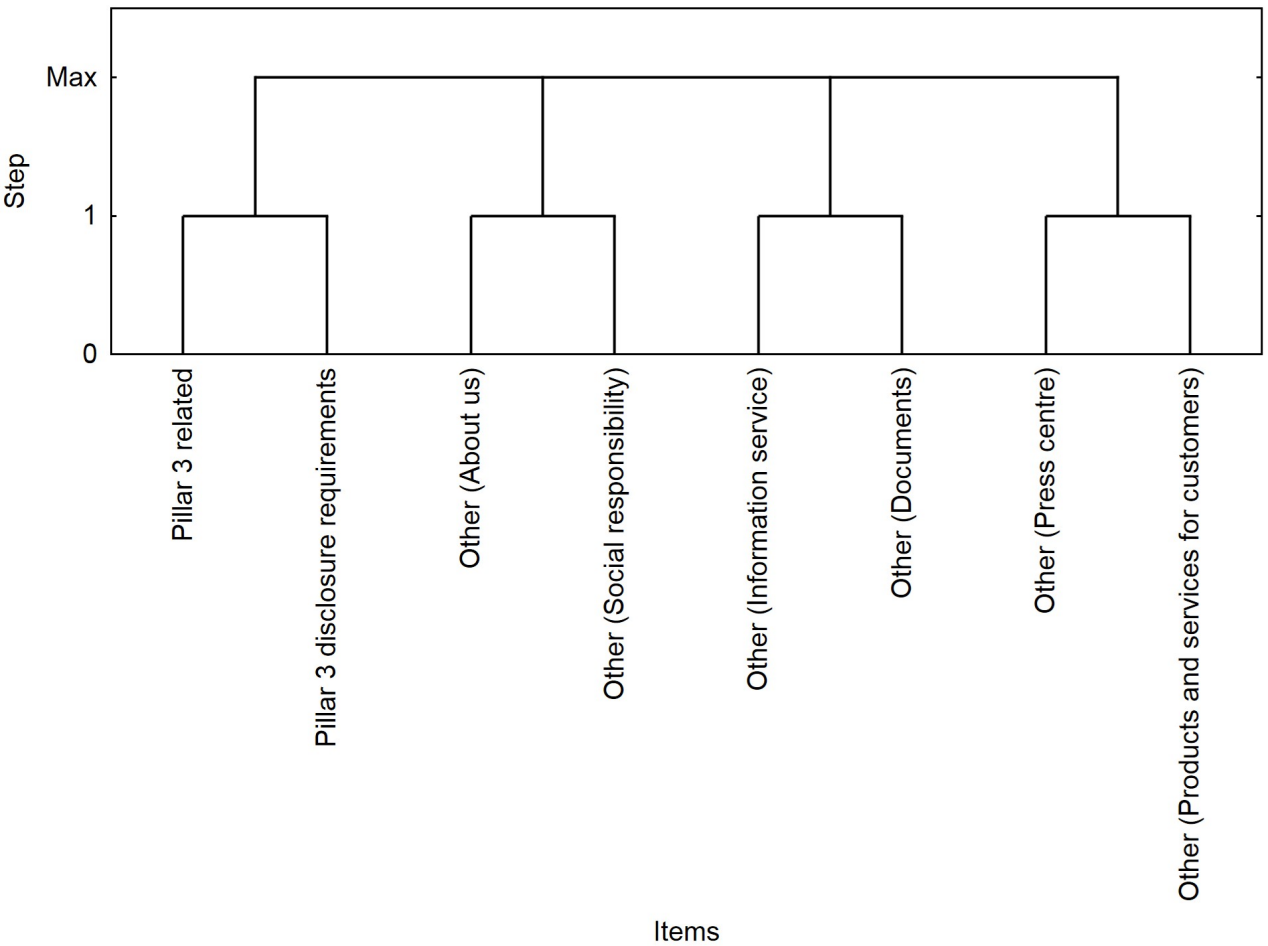
Visualization of segments in the fourth quarter.

The segmentation results (Fig 9) confirm the results of the association analysis for the web part pairs (*/Pillar3 disclosure requirements/*, */Pillar3 related/*), (*/Other—About* us*/*, */Other—Social responsibility/)* and (*/Other—Information Service/*, */Other—Documents/*), where a positive correlation was identified (*lift* > 1). Part of the sessions was characterized by a visit to both Pillar 3 web parts, where the highest level of interest was achieved (*lift* = 59).

#### Results summary

The results reveal that during the year 2018 the most visited web parts pair was (*/Other—Products and services for customers/*, */Other—Information Service/*). The highest level of positive correlation was achieved by a pair (*/Pillar3 disclosure requirements/*, */Pillar3 related/*). The results revealed that stakeholders showed higher interest in Pillar 3 related information, Annual reports, Information on Group than in Pillar 3 disclosure requirements. This could imply that the stakeholders found important content in these categories that are not published in Pillar 3 disclosures.

We evaluated the patterns of stakeholder behaviour in terms of time (years and quarters) to verify the presence or absence of a time trend and seasonality in the behaviour of stakeholders in relation to Pillar 3 information. For this purpose, we evaluated the proportion of occurrence of patterns with Pillar 3 information from all possible combinations of the examined web parts.

In the case of years (Table 4a–4d) for all quarters (Q1: *Q* = 4.666667, *df* = 2, *p* = 0.096974; Q2: *Q* = 3.600000, *df* = 2, *p* = 0.165301; Q3: *Q* = 2.000000, *df* = 2, *p* = 0.367881; Q4: *Q* = 0.000000, *df* = 2, *p* = 1.000000), the null hypotheses are not rejected, i.e. the occurrences of frequent item sets of web parts related to Pillar 3 do not depend on the year.

**Table 4:** Homogeneous groups for the occurrence of frequented item sets of web parts related to Pillar 3 for the quarter a) Q1, b) Q2, c) Q3 and d) Q4.

Year	Percent	1
18Q1	25.00%	****
17Q1	30.56%	****
16Q1	33.33%	****
17Q3	33.33%	****
18Q3	36.11%	****
16Q3	38.89%	****
18Q2	25.00%	****
17Q2	33.33%	****
16Q2	33.33%	****
17Q4	36.11%	****
18Q4	36.11%	****
16Q4	36.11%	****

Note:

****—homogeneous groups (p > 0.05).

One homogeneous group (16QX, 17QX, 18QX) was identified for each quarter (Table 4a–4d) based on the average occurrence of the found frequent item sets of web parts related to Pillar 3 information (*p* > 0.05). The results correspond to our previous findings [7] on the absence of a time trend in the years after the crisis.

In the case of the examined web parts related to Pillar 3 (Table 5c), no statistically significant differences (2018: *Q* = 7.714, *df* = 3, *p* = 0.0523) were identified between the individual quarters of 2018. The most frequent item sets related to Pillar 3 in 2018 (Table 5c) were identified in the third and fourth quarters (< 40%), the least in the first and second quarters (< 30%). Similarly, seasonality in the interest of Pillar 3 information (Table 5a and 5b) was not identified in 2016 and 2017 (2016: *Q* = 1.737, *df* = 3, *p* = 0.6288; 2017: *Q* = 0.857, *df* = 3, *p* = 0.8358), which confirms the established trend identified in the years after the crisis 2012–2015 (*p* > 0.05) [7], i.e. in the years after the crisis, no seasonality was identified in accesses to Pillar 3 information.

**Table 5:** Homogeneous groups for the occurrence of frequented item sets of web parts related to Pillar 3 for the year a) 2016, b) 2017 and c) 2018.

Quarter	Percent	1
16Q1	33.33%	****
16Q2	33.33%	****
16Q4	36.11%	****
16Q3	38.89%	****
17Q1	30.56%	****
17Q3	33.33%	****
17Q2	33.33%	****
17Q4	36.11%	****
18Q2	25.00%	****
18Q1	25.00%	****
18Q4	36.11%	****
18Q3	36.11%	****

Note:

****—homogeneous groups (p > 0.05).

## Conclusion and contribution of the work

The disclosure of commercial banks is of particular importance for their stakeholders. In the process of the search for meaningful Pillar 3 disclosures as a direct channel for market discipline implementation, it is important to take into account a lot of factors, which influence their quality. These factors are transparency, accuracy, timing, which serves as a background for meaningful, comparable, and sufficient disclosures to deliver relevant information to key market participants. However, if market discipline should be effective it requires the relevant interest of key market participants in regulatory required Pillar 3 disclosed information. In our research, we studied these interests in commercial banks operating in CEE country. We found out that in studied banks single interest in regulatory required Pillar 3 information was very low and not equally intensive interest in regulatory required timing—quarters of the year.

In the first analysed bank, from 2009 to 2015, the most visited were web parts */Group/* and */Pillar3 Q-terly Info/*. The pairs identified with high interest were */Group/*, */Pillar3 Q-terly Info/* and */Annual Reports/*, */Pillar3 Q-terly Info/* in these years. Generally, stakeholders were interested in Pillar 3 disclosures (regulatory and accounting) mainly in the first quarter of the year. It is important to note, that after turbulent times in 2009 the interest of the stakeholders in disclosed information steadily decreased in the analysed first commercial bank in Slovakia.

In the second analysed bank, the results show that in all four quarters of 2018 the pair (*/Other—Products and services for customers/*, */Other—Information Service/*) was the most visited web parts with the highest *support*. Additionally, the highest level of positive correlation was achieved by a pair (*/Pillar3 disclosure requirements/*, */Pillar3 related/*). Moreover, the results of these analyses suggest that stakeholders expressed higher interest in Pillar 3 related information, Annual reports, Information on Group (financial reports, annual reports, information about the group, rating, general shareholder meeting, emitent prospects) than in Pillar 3 disclosure requirements. It can also indicate that these categories contain important content for web users, which is not published in Pillar 3 disclosures. Similar findings were achieved for 2016 and 2017.

The assumption about the continuation of the established trend after the gradual disappearance of the consequences of the financial crisis from 2012–2015 [7], which is characterized by a lack of interest in Pillar 3 information, was confirmed. It was further confirmed that the behaviour of stakeholders in relation to the published Pillar 3 information no longer shows any time trend or seasonality.

We saw an increased interest in this information only in the years of the global financial crisis and in the immediate aftermath of the crisis (2009–2011). The following years (2012–2018) were characterized by an only low interest in this information and based on data on the use of the web portals of two commercial banks, we did not identify any time trends or seasonality in the behaviour of stakeholders in relation to Pillar 3 information. Previous revisions of Pillar 3 by regulatory authorities have not had a significant impact on increasing interest in this information.

The results suggest that market discipline mechanisms should start to be ready and operate efficiently, particularly during turbulent times. If the commercial banking institutions owned by foreign shareholders in CEE countries are not ready to disclose information satisfactorily and on time it can cause losses related to the reputational risk of the bank. These results also suggest that changes in the Pillar 3 disclosures are inevitable to bring relevant and meaningful information to stakeholders which are key for this type of institution. The stability of the financial market is the most important goal of the regulators since the severe market turbulence, which influenced the whole financial system. Therefore, market discipline mechanism implementation should take into account balancing between its economic costs and benefits and should adequately respond to its current challenges. It is important to note, that the weaknesses of the Pillar 3 disclosures are present, but their advantages are undeniable due to the focus of the majority of theoretical studies on their positive aspects, as a beneficial tool for the enhancement of effective market discipline mechanism.

### Contribution to the improvement of the effective market discipline in commercial banks operating in CEE countries

In our research, we have identified areas of improvement, which can increase the interest of the stakeholders in the Pillar 3 disclosures, and we summarize them as the following recommendations:

- To improve standardisation, meaning harmonisation of national authorities’ disclosure requirements and disclosure requirements on EU level (Pillar 3 and national requirements).
- To increase comparability of disclosures by creating one common template (visually prescribed tables) ideally created by regulators, to implement uniformity.
- To decrease the frequency of the Pillar 3 disclosures, due to the low interests of the stakeholders in quarterly disclosures.
- If quarterly disclosures are applied to decrease the amount of the disclosed information could be beneficial and to differentiate extend of the annual disclosures (more information) in comparison to a quarterly one.
- To include information areas (either on an obligatory or voluntary basis), in which stakeholders are interested (business behaviour of the institution, strategy, rumours, structure, ownership, mission, values), which influence the risk position of the institution.
- Regulators should assure compliance with the obligatory required information—mainly to restrict the institutions’ omission of the required information without any indication of the reasons.
- To impose rules for the location of the disclosed documents, which should be in an identifiable section of the web page.
- Obligation to use English as a unified language for disclosures.

The stability of the financial market is the most important goal of the regulators since the severe market turbulences influenced the whole financial system. Therefore, market discipline mechanism implementation should take into account balancing between its economic costs and benefits and should adequately respond to its current challenges. It is important to note, that the weaknesses of the Pillar 3 disclosures are present, but their advantages are undeniable as a beneficial tool for enhancement of effective market discipline mechanism.

The future work will be focused on the verification of our findings particularly concerning the effectiveness of published and revised information in the period of the COVID-19 pandemic crisis.

### Research limitations

Our research has a few limitations. Importantly, these limitations are concerning the nature of the data and its characteristics. The extraction of the data also has deficiencies in the redundancy of the data, which means that the data source has also been aimed at customers of the bank web portal, who do not preferentially look for Pillar 3 data to assess the bank’s risk profile. Importantly, customers of the bank are also an important group of targets of market discipline implementation, but they are not a direct source of market discipline enhancement. Although these deficiencies in the data might have caused some statistical deviations, we consider them as minor deviations with weak influence on overall research results as customers of the bank are also part of the market discipline.
